# Between-Session Reliability of Strength- and Power-Related Variables Obtained during Isometric Leg Press and Countermovement Jump in Elite Female Ice Hockey Players

**DOI:** 10.3390/sports11050096

**Published:** 2023-04-29

**Authors:** Henrik Petré, Niklas Psilander, Hans Rosdahl

**Affiliations:** Department of Physiology, Nutrition, and Biomechanics, The Swedish School of Sport and Health Sciences, 11486 Stockholm, Sweden; henrik.petre@gih.se (H.P.);

**Keywords:** reproducibility, isometric peak force, fatigue, performance, injury prevention

## Abstract

Isometric leg press (ILP) and countermovement jump (CMJ) are commonly used to obtain strength- and power-related variables with important implications for health maintenance and sports performance. To enable the identification of true changes in performance with these measurements, the reliability must be known. This study evaluates the between-session reliability of strength- and power-related measures obtained from ILP and CMJ. Thirteen female elite ice hockey players (21.5 ± 5.1 years; 66.3 ± 8.0 kg) performed three maximal ILPs and CMJs on two different occasions. Variables from the ILP (peak force and peak rate of force development) and CMJ (peak power, peak force, peak velocity, and peak jump height) were obtained. The results were reported using the best trial, an average of the two best trials, or an average of three trials. The intraclass correlation coefficient (ICC) and coefficient of variation (CV) were high (ICC > 0.97; CV < 5.2%) for all outcomes. The CV for the CMJ (1.5–3.2%) was lower than that for the ILP (3.4–5.2%). There were no differences between reporting the best trial, an average of the two best trials, or an average of the three trials for the outcomes. ILP and CMJ are highly reliable when examining strength- and power-related variables in elite female ice hockey players.

## 1. Introduction

Isometric leg press (ILP) and countermovement jump (CMJ) are two commonly used measurements among practitioners and researchers to measure strength and power characteristics in the lower body. These measurements are likely popular because they are considered safe, informative, and easy to perform in terms of technique. In addition, they provide common useful variables such as peak force (PF), rate of force development (RFD), peak power (PP), peak velocity (PV), and peak jump height (PH). Testing of strength- and power-related variables has important implications in different public health contexts. For example, in the elderly, a progressive loss of strength and power is observed, especially in the leg musculature [1] which is associated with an increased injury risk and reduced general health maintenance [2,3]. Aside from the health perspective, strength and power are considered to be two of the most important performance demands for success in several sports [4]. Hence, sports scientists and practitioners continuously perform physical tests of the players to capture and analyze fluctuations in these variables to enable informative decisions when planning training. These variables can be used with the purpose to assess association with specific sports performance measures (i.e., linear sprint speed or change of direction), to monitor neuromuscular fatigue, or to assess progress and preparedness for return to sport during rehabilitation [5]. Thus, these variables have important implications for both sports performance and health maintenance in athletes. To enable differentiation between variation in the measurement method and true changes in performance, the reliability of these methods must be known. This includes measures of test–retest reliability and intervals/limits for the detection of a true change (sensitivity). This is particularly important when testing elite athletes as the adaptations in this population after training are small. Since biological variance is suggested to be the main source of variance [6] and differences in force and force–time characteristics exist between athletes from different sports and between genders [7], it is vital that the reliability of these methods is known in athletes from different sports. This is particularly important in strength- and power-dependent sports where these measurements are often applied.

Ice hockey is a strength- and power-dependent sport including high-intensity sprint bouts interspersed with recovery periods at lower intensity and passive rest periods between shifts and periods. Given the stop-and-start nature of the game, players repeatably need to develop high force and power [8]. This is supported by studies investigating player characteristics, revealing strong and powerful athletes [9], and studies investigating the relationship between skating capacities and strength [10,11] and that between skating capacities and vertical jump performance [12,13,14]. For these athletes, strength and power are continuously measured, and strength training as well as vertical jump training are integrated parts of their weekly training routine. Knowledge of the reliability of the methods used to assess strength- and power-related variables in these athletes is therefore particularly important. Strength- and power-related variables have been investigated and considered reliable during ILP [15,16] and CMJ [17,18,19] in male athletes and in a mixed group of team sport athletes with different training background and gender. However, no study has examined the reliability of these measurements in elite female ice hockey players. The primary aim of this study was therefore to establish the variation between strength- and power-related measures obtained from the ILP and CMJ measurements taken on different days in elite female ice hockey players.

It has been proposed that the number of trials used to extract values for the final analysis can affect the results (best trial or an average of more trials in the same session) [20]. However, there is a lack of research examining this in female ice hockey players. The secondary aim of this study was therefore to analyze whether reporting the best trial, an average of the two best trials, or an average of three trials affects the results.

## 2. Materials and Methods

### 2.1. Experimental Design

A study with a single-group repeated-measure design was performed in which participants performed ILP followed by CMJ at the same time of the day, on two different occasions, separated by 24 h. Three maximal effort trials were allowed for each exercise (ILP and CMJ) on each of the two occasions. To determine the reliability, paired sample *t*-test, intraclass correlation coefficient (ICC), coefficient of variation (CV), and limits of agreement (LOA) were used. The participants were asked to avoid strenuous activity >48 h before baseline testing, refrain from strenuous lower body training between the two testing occasions, and replicate the nutrition intake on each testing day. Every testing session started with a standardized warm-up including 10 min of continuous cycling (Monark 894, Vansbro, Sweden) at 120 W followed by 2 min of dynamic stretching. The ILP was performed first and followed by the CMJ.

### 2.2. Participants

Thirteen female elite-level ice hockey players (age 21.5 ± 5.1 years, body height 167.6 ± 4.1 cm, body mass 66.3 ± 8.0 kg) volunteered to participate in the study and all participants completed all trials. The participants were highly trained elite ice hockey players from the highest ice hockey league in Sweden, one of the highest-ranked leagues in the world. They were only recruited if they had an average weekly training volume of 8–10 training sessions and included countermovement jump and maximal strength training for the lower limbs (>80% of 1 RM) as a part of their normal training routine. All participants were familiarized with the test protocol by performing three familiarization sessions before the intervention. To minimize the impact of the menstrual cycle on performance, none of the participants performed any of the experimental sessions during the ovulation phase of the menstruation cycle as this period seems to affect isometric maximal strength performance [21]. The participants were fully informed about the study, risks, and benefits of participation, made a health declaration, and provided their written informed consent before participation. The study was approved by the Regional Ethical Review Board in Stockholm (Dnr 2022/01818-01) and performed according to the principles outlined in the Declaration of Helsinki.

### 2.3. Test Protocols

#### 2.3.1. Isometric Leg Press

Peak force and rate of force development (RFD) were determined with ILP using an IsoMed 2000 dynamometer (D&R Ferstl GmbH, Hemau, Germany) with multi-joint leg press-system (IsoMed 2000 linear module) equipped with force plates and a sampling frequency of 2000 Hz. The analog signal from the force plates was converted into a digital signal using a CED power 1401 data acquisition system (version 7.0, Cambridge Electronic Design, Cambridge, UK) and collected into Spike 2 software (version 7.09a, Cambridge Electronic Design, Cambridge, UK). The dataset was low pass filtered using a Butterworth filter with a cut-off frequency of 10 HZ and transported to Matlab (version R2020a, The Mathworks, Inc., Natick, MA, USA) for further data processing and calculation of the outcome variables (see below).

**Figure 1:**
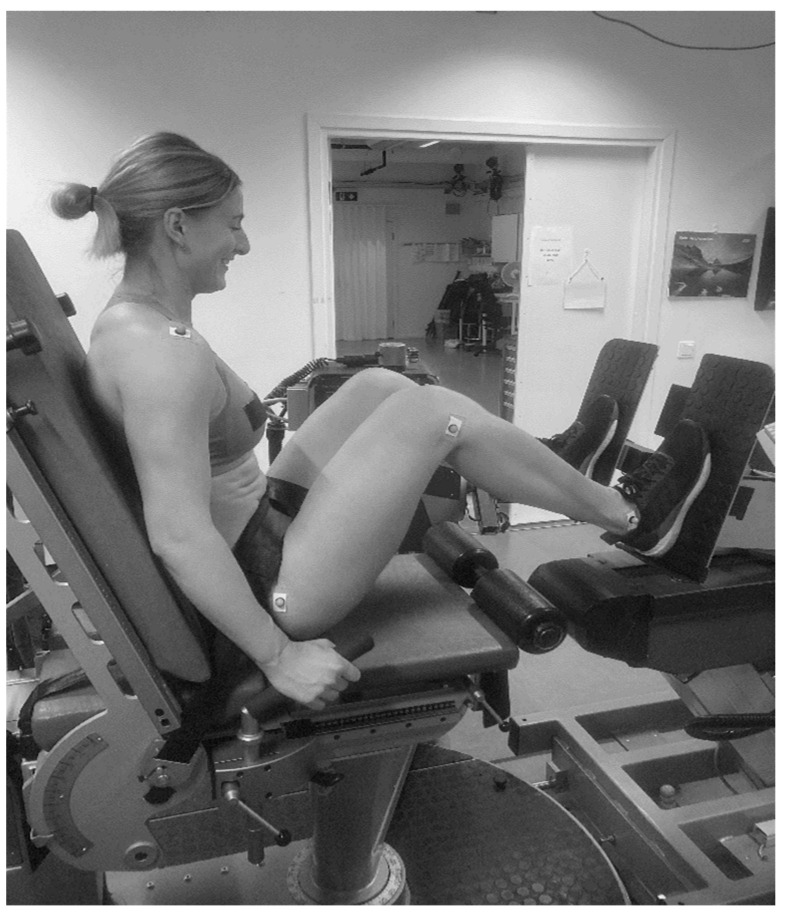
Position during testing of ILP.

The dynamometer chair was positioned at a hip angle of 70°, knee at 90° knee, and ankle at 15° (Figure 1). As reference points for correct knee angle, trochanter major, lateral femoral epicondyle, and malleolus were used, and knee angle was measured with a goniometer. To stabilize the set-up, adjustable straps were placed over the hip, and the participants were instructed to hold on to the side handles situated on the lateral of the chair during the test. All participants were informed to perform all testing and familiarization sessions with the same shoes and with the same distance between feet to ensure standardization of the test procedure. The participants were instructed to apply 300 N of pretension before starting the explosive contraction to avoid force oscillation. Before the first 1 RM efforts, each participant performed 3 submaximal trials. Each participant was allowed three maximal trials. The maximal trials were initiated by a countdown of “3, 2, 1, GO”, and the participants were instructed to press as hard and fast as possible against the force platforms. All participants were verbally encouraged during each trial to ensure maximal effort for 3 s, and a 60 s rest period was provided between each trial.

Peak force was recorded as the highest force value obtained from the force curve. The rate of force development was calculated as the maximal slope of the force–time curve (∆force/∆time) within a 50 ms time window from the onset of contraction [22]. The onset of contraction was set to 2.5% of the difference between peak force and baseline force [23]. Baseline force was defined as the 1 s mean with the lowest standard deviation (SD) of 2.5 s prior to when peak force was obtained. The maximal peak force and RFD from the three trials, the average of the two best trials, and the average of all three trials were used for further analysis for each test session.

#### 2.3.2. Countermovement Jump

**Figure 2:**
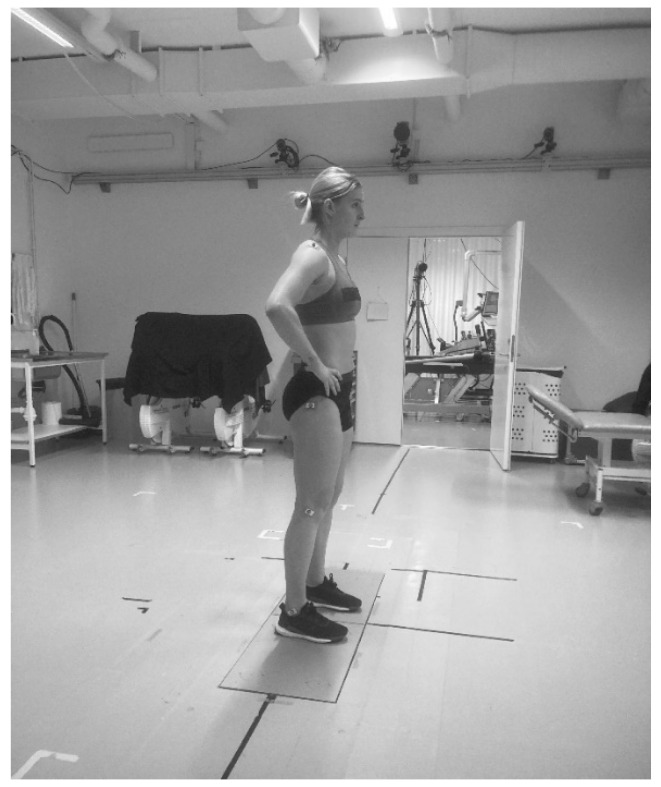
Starting position during the countermovement jump.

Maximal CMJ was performed on force plates (Kistler instrument corporation, Winterthur, Switzerland, model. 9281EA) (Figure 2). Zero offset was performed by unloading the force plates prior to each trial. Matlab (version R2020a, The Mathworks, Inc., Natick, MA, USA) was used to process the data and for the calculation of the outcome variables (see below). Three submaximal CMJs were performed before the first 1 RM CMJ as a part of the warm-up. Each participant performed three maximal 1 RM trials. The participants were instructed as follows: (1) to place their hands on the hips (akimbo) during the jumps, (2) to self-select their jump depth, (3) to stand still on the platforms for three seconds before initiating the jump, (4) to perform the CMJ as a continuous movement without pausing, (5) to try to jump as high as possible every trial, and (6) to land on the platform and avoid displacement in the lateral or frontal plane. Peak jump height was calculated using the impulse–momentum method as previously described [24]: peak jump height = velocity at takeoff^2^/2 g, in which g = 9.81 m/s^2^. To compute takeoff velocity, acceleration was first computed by subtracting body weight from the vertical ground reaction force (vGRF) and subsequently dividing it by body mass. Velocity was computed by integrating acceleration using the cumtrapz function in Matlab. Takeoff was defined as the instant when the force plate was unloaded. Peak force, peak velocity, and peak power were defined as the highest value obtained in each of the outcomes before takeoff. Power was computed by multiplying vGRF by velocity. Each trial was separated by a 60 s rest period and for each outcome variable, the value from the best trial from the three trials, the average of the two best trials, and the average of all three trials were used in the final analysis for each session.

### 2.4. Statistical Analyses

The statistical analysis was performed in SPSS (version 27, IBM Corporation, Armonk, NY, USA). The normality of the data was checked with the Shapiro–Wilk test. To control for systematic bias, a paired sample *t*-test was performed. The statistical significance for all analyses was set to *p* < 0.05. To measure relative reliability, the intraclass correlation coefficient (ICC2, k) was used [25,26]. The threshold for reliability using the ICC was considered as follows: moderate reliability (0.50–0.75), good reliability (0.75–0.90), and excellent reliability (>0.90) [27]. To measure absolute reliability, the coefficient of variation (CV) and Bland and Altmann’s 95% limits of agreement (LOA) were used. CV was calculated as CV% = (SE of the between-day difference/mean) × 100 [6]. Microsoft Excel (version 2102) was used to calculate the 95% LOA in the Bland and Altmann plot and for the 95% confidence interval for the CV. To compare the best trial, an average of the two best trials, or an average of three trials, a repeated-measure ANOVA was used with the mean differences for the six outcomes as dependent variables (ILP PF, ILP RFD, CMJ PP, CMJ PF, CMJ PV, and CMJ PH) and the three methods as independent variables (best trial, an average of the two best trials, and an average of three trials).

## 3. Results

**Figure 3:**
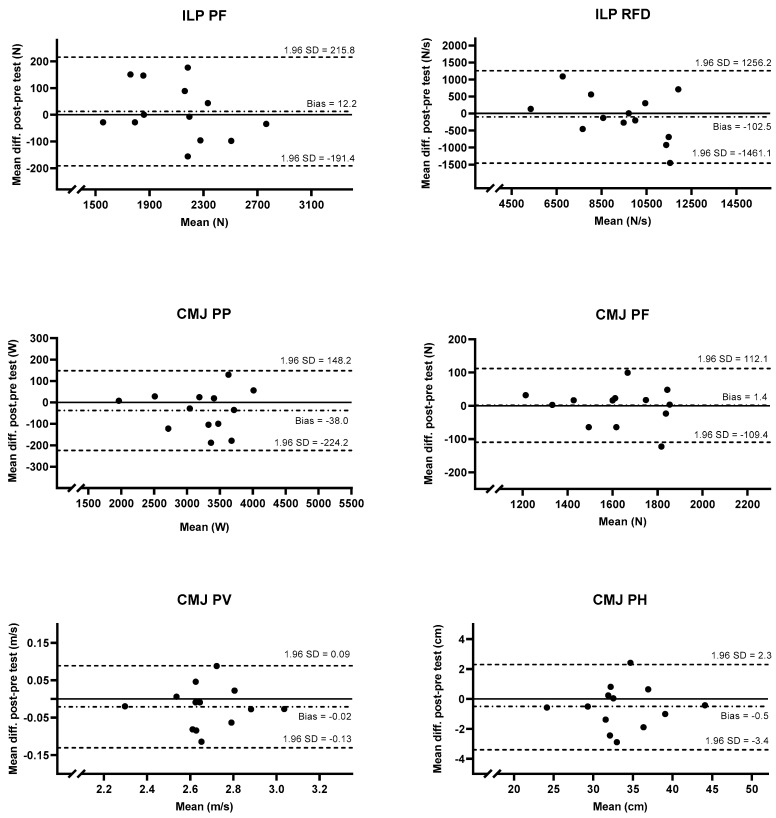
Bland–Altmann plots presenting test–retest for ILP PF, ILP RFD, CMJ PP, CMJ PF, CMJ PV, and CMJ PH using the best trial. The dashed line in the middle is the mean bias, and the upper and lower dashed lines are the upper and lower 95% LOA. The solid line is zero.

The results from the strength and power measurements and the reliability analysis are presented in Table 1 and Figure 3. The Shapiro–Wilk test showed a normally distributed dataset (*p* > 0.05). The paired sample *t*-test showed no significant difference between the test sessions for any of the strength- and power-related variables measured with ILP and CMJ (*p* > 0.05). Intraclass correlation coefficients were excellent for all included variables (ICC = 0.97–0.99). In general, the CMJ measurements resulted in lower CV compared with the ILP measurements (1.5–3.2% vs. 3.4–5.2%). The lowest CV was observed for CMJ PV (1.5%), and the highest was observed for ILP peak RFD (3.7–5.2%) (Table 1). The repeated-measure ANOVA showed no significant difference between the best trial, an average of the two best trials, and the average of the three best trials for any of the outcomes (ILP PF, *p* = 0.250; ILP RFD, *p* = 0.206; CMJ PP, *p* = 0.890; CMJ PF, *p* = 0.652; CMJ PV, *p* = 0.265; CMJ PH, *p* = 0.354).

## 4. Discussion

This is the first study investigating the between-session reliability of ILP and CMJ in elite female ice hockey players. The results show that ILP (peak force and RFD) and CMJ (PF, PP, PV, PH) display high reliability between sessions (ICC > 0.97 and CV < 5.2%) in female elite ice hockey players. The present results also show that CMJ is the most reliable measurement for these athletes (ICC > 0.97 and CV < 3.2%). Using the best trial, the average of the two best trials, or an average of three trials does not affect the result for any of the outcomes.

ICC for both ILP and CMJ showed excellent relative reproducibility with the lowest bound of the 95% confidence interval above the threshold for excellent reliability (>0.90). ICCs for ILP and CMJ were almost identical (0.97–0.99 and 0.97–0.99), while the CV appears to be lower for the CMJ outcomes (CV = 1.5–3.2%) compared to the ILP (CV = 3.4–5.2%) regardless of the use of the best trial, an average of two best trials, or an average of three trials. These findings might be explained by between-subject variation in the magnitude of the score in the measured variable. In contrast to CV, ICC is dependent on the between-subject variation and heteroscedastic data [28,29]. If the variation between participants is high, ICC can be high even though the within-subject variation is high [26,28]. A high range of scores between participants was observed for both ILP PF (1531–2746 N) and ILP RFD (5129–11,445), and we, therefore, believe between-subject variation might have affected the results for ICC. This observation is supported by the Bland–Altmann plots in Figure 3 displaying the highest proportional bias (heteroscedasticity) for the ILP PF and ILP RFD measurements. In contrast to the plots for the CMJ, the plots for ILP indicate that higher scores seem to lead to a negative bias while lower scores lead to a positive bias (Figure 3). What appears to be a higher between-subject variation in ILP measures compared to CMJ might be explained by the difference in the direction of force applied between the tests. In contrast to CMJ, the participants during ILP does not lift their body weight, which would be an advantage for heavier athletes and allow for a greater range between individuals with different body weights. A large variation in body weight was observed (53.0–77.1), which is normal when including ice hockey players [30] and probably explains the higher between-subject variation in ILP compared with CMJ.

That ILP displayed the highest CV was not expected since ILP is performed in a fixed set-up with superior standardization compared to CMJ which is more dependent on technical jumping skills. The reason for these results might be that all participants were skilled in CMJ since this exercise was included in the players’ daily training routine while the isometric leg press was not. In addition, in contrast to CMJ, the average of three trials seems to give the most reliable value when extracting the results for the final analysis for the ILP. It is therefore possible that elite-level female ice hockey players need more trials during ILP measurements than during CMJ. Regardless, these results highlight the importance of including CV when measuring reliability during ILP testing as a large variation in the magnitude of the score is expected and proportional bias is common in sport science [29].

ILP has been used in several studies for measuring maximal isometric strength [31,32,33,34]. Despite this, surprisingly few studies have established the reproducibility of this measurement. Recently, Gaspari et al. [15] reported good reliability of ICC = 0.88 but with the lowest 90% confidence interval bound only at moderate reliability (ICC = 0.67) in eleven female, high-level athletes from different sports. The different results between this study and the present study could be attributed to methodological differences. In the study by Gaspari et al. [15], a similar standardization was used in terms of equipment and visual feedback. However, standardization of joint angles (knee, 120° compared to 90°; hip, 100° compared to 70°) was different, as were the level of pre-tension (128 N compared to 300 N) and the number of familiarization sessions (one session compared to three). In addition, the present study included highly trained individuals with the same training background, whereas the study by Gaspari and colleagues included a combination of gymnasts, basketball players, volleyball players, and track and field athletes with lower training status and training frequency. Therefore, to obtain good reliability when measuring isometric strength in female athletes, we recommend applying the standardization used in the present study and considering athletes with a similar training background and performance level. Hence, considering the importance of these measurements in public health contexts and that few studies have investigated the reliability of the ILP, the standardization in the present study could be recommended for other populations as well (i.e., older adults).

In a nonathletic population, previous studies have reported CV values of <6.0% in young participants [35] and <9.8% in elderly participants [36] when examining the reliability of strength- and power-related variables during CMJ on force plates. With regard to an athletic population, CV values between 2.0 and 9.8% have been reported [17,18,19]. The high reliability observed in the present study might be explained by the participants’ training background and performance level. In contrast to the previous studies, the present study included elite athletes with similar training background involving CMJ training on a regular basis. Interestingly, and similar to findings from earlier studies, both in a nonathletic population [36] and in an athletic population [17,19], CMJ jump height was the outcome with the lowest reproducibility among the measured outcomes from the jump testing. This might be because this outcome is not measured directly by the force plates, but instead calculated through a mathematical assumption. Practitioners and athletes assessing performance in peak jump height in female ice hockey players may consider a change greater than +2.3 to −3.4 cm as a true change in performance when reporting the results using the best trial (with 95% confidence) (Figure 3).

Despite the novelty of the present findings, this study is not without limitations. The rest period between sessions (24 h) was relatively short. However, as the tests were performed at very low volume and the participants were highly trained elite-level athletes, we considered 24 h to be sufficient. The experimental session was performed within the follicular or the luteal phase of the menstruation cycle. It has been proposed that fluctuations in hormones during the menstruation cycles can affect neuromuscular function and fatigability [37]. However, whether this has an impact on strength and power measures is not fully elucidated. Peltonen et al. [21] demonstrated that despite an association between fatigue and levels of estrogen and progesterone and the difference between phases of the menstruation cycle, no difference in isometric maximal strength or dynamic power could be found between the phases. Since our experimental session was performed with only 24 h between the sessions, none of the participants moved between phases during the experimental sessions. In addition, the participants were elite athletes used to high training volumes during all phases of the menstruation cycle. We, therefore, consider the fluctuation of hormones during the menstruation cycle to have minimal impact on the results. In this study, we investigated a specific population, elite female ice hockey players, with very similar training backgrounds. Hence, the results for CMJ and ILP are not directly transferable to other populations and more studies are therefore warranted, especially on ILP reliability.

### Practical Applications

The findings from this study provide novel information on the test–retest reliability for elite female ice hockey players. This information could be useful for practitioners and researchers while making interpretations of the outcomes from ILP and CMJ measurements to adjust training to increase performance and prevent injury. Sports scientists and practitioners can confidently use the ILP peak force and rate of force development as well as CMJ peak power, peak force, peak velocity, and peak jump height to assess change in performance in female ice hockey players. When looking to identify a true increase (95% LOA) in performance between test sessions, practitioners should look for changes >215.8 N ILP PF, >1256.2 N/s ILP RFD, >148.2 W CMJ PP, >112.1 N CMJ PF, >0.09 m/s CMJ PV, and >2.3 cm CMJ PH when reporting the results using the best trial. Furthermore, when reporting the results, it appears that either the best trial, an average of the two best trials, or an average of the three trials can be used without reducing the reliability of the results.

## 5. Conclusions

The present study shows that ILP and CMJ are highly reliable measurements when examining strength- and power-related variables in elite female ice hockey players. Surprisingly, the highest reproducibility between sessions was found for the outcomes obtained from the more technical skill-dependent CMJ. Moreover, the choice of reporting the best trial, an average of the two best trials, or an average of the three trials did not affect the results.

## Figures and Tables

**Table 1:** Between-session reliability for ILP and CMJ.

Variable	Session 1		Session 2		Change		*p*	ICC (95% CI)		CV% (95% CI)	
	Mean	SD	Mean	SD	Mean	SD					
ILP											
Peak force max (N)	2104	354.2	2116	322.6	12.2	104	0.68	0.98	(0.93–0.99)	3.48	(−3.34–10.31)
Peak force mean 2 (N)	2080	353.0	2076	309.8	−3.72	99.4	0.90	0.98	(0.93–0.99)	3.38	(−3.25–10.01)
Peak force mean 3 (N)	2042	347.3	2050	304.7	8.30	97.2	0.76	0.98	(0.93–0.99)	3.36	(−3.23–9.94)
											
Peak RFD max (N/s)	9415	2035	9312	1828	−103	693	0.60	0.97	(0.90–0.99)	5.23	(−5.03–15.50)
Peak RFD mean 2 (N/s)	9177	1987	9103	1764	−73.5	578	0.66	0.98	(0.93–0.99)	4.47	(−4.29–13.24)
Peak RFD mean 3 (N/s)	8881	1902	8906	1725	25.3	459	0.85	0.99	(0.95–0.99)	3.65	(−3.51–10.81)
CMJ											
Peak power max (W)	3252	562.5	3214	559.7	−38.0	95.0	0.18	0.99	(0.98–0.99)	2.08	(−1.99–6.15)
Peak power mean 2 (W)	3229	560.2	3194	564.0	−35.7	78.6	0.13	0.99	(0.98–0.99)	1.73	(−1.66–5.12)
Peak power mean 3 (W)	3207	554.4	3170	561.5	−37.2	81.7	0.13	0.99	(0.98–0.99)	1.81	(−1.74–5.36)
											
Peak force max (N)	1620	204.3	1621	212.3	1.37	56.5	0.93	0.98	(0.94–0.99)	2.47	(−2.37–7.30)
Peak force mean 2 (N)	1605	207.4	1608	210.8	2.72	57.3	0.15	0.98	(0.94–0.99)	2.52	(−2.42–7.46)
Peak force mean 3 (N)	1594	204.7	1598	207.9	4.29	58.2	0.14	0.98	(0.94–0.99)	2.58	(−2.48–7.64)
											
Peak Velocity max (m s ^−1^)	2.69	0.18	2.67	0.18	−0.02	0.06	0.20	0.97	(0.92–0.99)	1.47	(−1.42–4.36)
Peak Velocity mean 2 (m s ^−1^)	2.68	0.18	2.66	0.18	−0.02	0.06	0.16	0.97	(0.92–0.99)	1.47	(−1.41–4.34)
Peak Velocity mean 3 (m s ^−1^)	2.67	0.18	2.65	0.19	−0.03	0.06	0.12	0.97	(0.91–0.99)	1.51	(−1.45–4.46)
											
Peak height max (cm)	34.0	4.9	33.4	4.9	−0.5	1.4	0.21	0.98	(0.93–0.99)	3.02	(−2.90–8.94)
Peak height mean 2 (cm)	33.7	4.9	33.1	5.0	−0.7	1.5	0.87	0.97	(0.91–0.99)	3.20	(−3.07–9.47)
Peak height mean 3 (cm)	33.4	4.8	32.8	5.0	−0.7	0.2	0.80	0.97	(0.91–0.99)	3.24	(−3.11–9.60)

ILP, isometric leg press; CMJ; countermovement jump; RFD, rate of force development; ICC, intraclass correlation coefficient; CV, coefficient of variation; CI, confidence interval; *p*, *p*-value from paired *t*-test.

## Data Availability

All data generated from this study can be found in the article.
